# The catalytic tetrad of *Aedes aegypti* argonaute 2 is critical for the antiviral activity of the exogenous siRNA pathway

**DOI:** 10.1016/j.jbc.2025.108332

**Published:** 2025-02-19

**Authors:** Krittika Dummunee, Rhys H. Parry, Lars Redecke, Margus Varjak, Benjamin Brennan, Alain Kohl, Melanie McFarlane

**Affiliations:** 1 MRC-University of Glasgow Centre for Virus Research, University of Glasgow, Glasgow, UK; 2 School of Chemistry and Molecular Biosciences, The University of Queensland, St Lucia, Australia; 3 University of Lübeck, Institute of Biochemistry, Lübeck, Germany; 4 Deutsches Elektronen Synchrotron (DESY), Photon Science, Hamburg, Germany

**Keywords:** RNA interference (RNAi), small-interfering RNA (siRNA), Argonaute, RNA virus, alphavirus, mosquito, antiviral

## Abstract

Viruses transmitted by biting arthropods, arboviruses, pose a significant global health and economic threat. Climate change is exacerbating this issue by expanding the range of disease-carrying vectors. Effective control of arbovirus transmission often relies on targeting the vectors, making it crucial to understand the interactions between the virus and its vector. The exogenous siRNA (exo-siRNA) pathway is a key antiviral defense mechanism in mosquitoes such as *Aedes aegypti*. Argonaute 2 (Ago2) is a central protein in this pathway, responsible for antiviral activity. While the PIWI domain of Ago proteins is known to mediate slicing activity, not all Ago proteins possess this slicing function. To understand the antiviral mechanism of Ago2 in *Ae. aegypti*, we aimed to confirm the presence of the catalytic tetrad, a group of amino acids known to be crucial for slicing activity. Here, we confirmed the tetrad (D740, E780, D812, and H950) in *Ae. aegypti* Ago2 and demonstrated its essential role in antiviral and siRNA pathway activity. Our findings show that the catalytic tetrad is necessary for the degradation of siRNA passenger strands. When the tetrad is absent, siRNA duplexes accumulate, leading to a loss of siRNA pathway function. This underscores the critical role of the tetrad in the antiviral defense mechanism of *Ae. aegypti*.

Arboviruses are a group of viruses that can be transmitted to, and cause disease in, susceptible hosts through the bite of an infected arthropod vector. These pathogens encompass several unrelated families of viruses, many of whom are of significant medical and economic importance. Most arboviruses are RNA viruses from the families Togaviridae and Flaviviridae and the class *Bunyaviricetes*. Significant examples include the alphavirus chikungunya virus (CHIKV), the flaviviruses dengue (DENV) and Zika (ZIKV) viruses, and Rift Valley fever virus (RVFV) from the bunyavirus family *Phenuiviridae*. Vectors responsible for the transmission of arboviruses include mosquitoes, midges, and ticks. In particular, the mosquito species *Aedes aegypti (Ae. aegypti)* is a critical vector for human-infecting arboviruses such as CHIKV, ZIKV, and DENV. With climate change increasing the geographical range of mosquito habitats, populations may increasingly be at risk from mosquito-borne diseases.

Arboviruses actively replicate in vectors such as mosquitoes, triggering an immune response to control the infection. In *Ae. aegypti* a critically important antiviral immune response is the exogenous small interfering RNA (exo-siRNA) response (1, 2, 3, 4, 5, 6). The exo-siRNA response is triggered by dsRNA, such as those generated as viral replication intermediates. Upon detection of dsRNA, an endonuclease (Dcr2) cleaves the dsRNA into virus-derived small-interfering RNAs (vsiRNAs) of mainly 21 nucleotides in length. These vsiRNAs are then transferred to Ago2 in the RNA-Induced Silencing Complex (RISC). Ago2 is a multi-functional protein that can bind the vsiRNAs, unwind the duplex, and degrade one of the siRNA strands, termed the passenger strand. Ago2 can then use the remaining strand, termed the guide strand, to locate and bind to complementary sequences present in viral genomes or mRNAs. This will result in the degradation of target RNA and subsequent “silencing” of viral replication. Overall the antiviral effects of the exo-siRNA pathway have been shown against arboviruses of multiple families (7, 8, 9, 10, 11, 12, 13, 14, 15, 16, 17, 18, 19, 20, 21, 22, 23, 24, 25, 26, 27, 28, 29, 30, 31, 32, 33, 34, 35, 36).

Ago2 is relatively well conserved across mosquitoes and functionally comparable to the Ago2 protein of the distant insect model *Drosophila melanogaster* which suggests critical conserved core functions. The Ago2 protein of *Ae. aegypti* contains a conserved PIWI domain that is essential for the endonuclease function of the Ago proteins, and the endonuclease activity of Ago proteins is determined by the presence of a catalytic tetrad in the RNAse H fold consisting of the amino acids DED(D/H/N) (37). However intriguingly, not all Ago proteins contain the DEDX sequence, for example, *Homo sapiens* Ago1, and therefore do not all possess endonuclease cleavage functionality (37). In this study, we aimed to determine whether the DEDX domain is present in *Ae. aegypti*-derived Ago2 and examine the relevance of this amino acid tetrad for antiviral activity. We show that the catalytic tetrad is in fact present across known mosquito Ago2 proteins and that it is essential for exo-siRNA activity and therefore the antiviral function of this effector protein. Finally, in the absence of DEDX domain, the Ago2 endonuclease activity is lost, mediated by the failure to degrade the passenger strand of the dsRNA duplex, thereby abrogating the antiviral function of Ago2.

## Results

### Identification of a DEDH catalytic tetrad in mosquito Ago2 proteins

**Figure 1:**
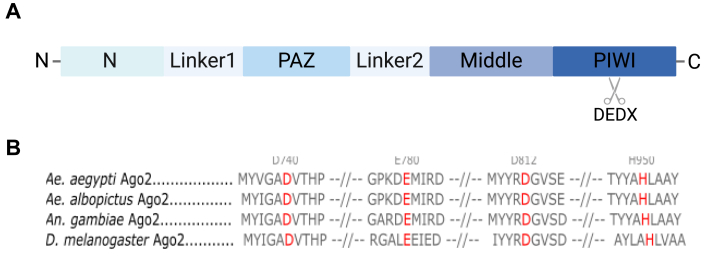
**Multiple sequence alignment of DEDH domain across vector mosquitoes.***A*, schematic diagram of the domain structure of Ago2. *B*, Ago2 sequences from *D. melanogaster* (UniProt ID: Q9VUQ5), *An. gambiae* (UniProt ID: A0A453Z118), *Ae. aegypti* (UniProt ID: C5J0H4) and *Ae. albopictus* (UniProt ID: I3VLA6) were aligned using Benchling (https://www.benchling.com/), and conserved amino acids D740, E780, D812 and H950 were identified relative to *D. melanogaster* Ago2 sequence.

Previous reports have identified a motif in the PIWI domain of Ago2 proteins which is well conserved across multiple species of bacteria and yeast and even mammalian species such as humans (37) (Fig. 1*A*). Here we assessed the presence and sequence of the catalytic tetrad across vector species of mosquitoes, *Ae. aegypti*, *Ae. albopictus*, *Anopheles gambiae* and the model organism *D. melanogaster*. Multiple sequence alignment analyses revealed the presence of a DEDH catalytic tetrad in all four species at amino acid positions D740, E780, D812, and H950 (Fig. 1*B*). An AlphaFold3 prediction of the three-dimensional structure of wild-type (wt) *Ae. aegypti* Ago2 shows that the DEDH residues are indeed surface exposed on the groove in the center of the protein and are biologically available, similar to that found in other species where the siRNA and mRNA are loaded and they can perform the slicing function of Ago2 (38) (Fig. S1*A*). Superposition of high-confidence structural models of the *Ae. aegypti* Ago2 PIWI domain comprising residues 719 to 963 and the corresponding part of *D. melanogaster* Ago2 (residues 947–1189) (Fig. S1*B*) further confirmed the structural conservation, particularly considering the positions of the DEDH residues composing the catalytic tetrad. Upon mutation of all four of the DEDH residues, no significant changes in the PIWI domain fold are predicted suggesting any functional differences displayed by this mutant are not due to protein misfolding (Fig. S1*C*). This can be attributed to the multiple sequence alignment (MSA)-based structure prediction approach of AlphaFold3, which averages out the impact of individual mutations on the structural model (39, 40). However, the DEDH residues are all located on the protein surface with side chains pointing away from the inner structure, which makes an impact of the mutations on the overall structure of the PIWI domain unlikely.

### Generation of cell lines expressing an Ago2 with a mutant catalytic tetrad

**Figure 2:**
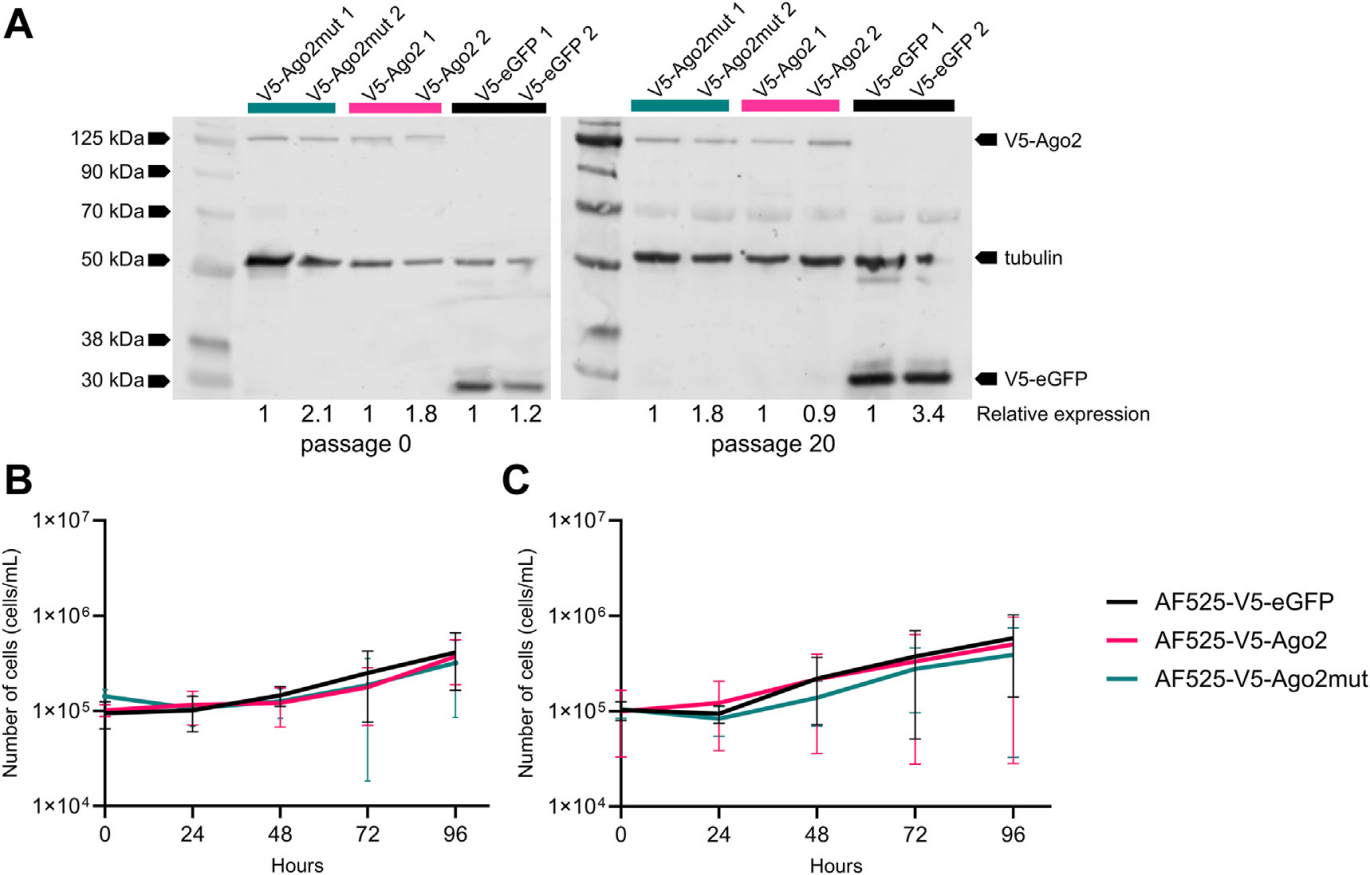
**Growth curve and stability analysis of the AF525-V5-eGFP, AF525-V5-Ago2, and AF525-V5-Ago2mut cell lines.***A*, Western blot analysis of cell lysates generated from the stable cell lines after initial generation (passage 0) and after long-term passage (passage 20). V5-tagged proteins are present in corresponding lanes confirming stable protein expression when probed with an anti-V5 antibody. Anti-tubulin was used as a loading control. Both cell line clones are indicated on the Western blot image. Relative protein expression levels are indicated below image. Protein expression levels were measured by calculating the density of each band normalised to the corresponding tubulin band and calculated relative to cell line 1. *B* and *C*, a total of 8 × 10^4^ cells were seeded into 24 well plates, and the number of cells present in the monolayer enumerated at the time points indicated for both clone one (*B*) and clone two (*C*) of each stable cell line. Points represent the average ± SD of three independent experiments.

The CRISPR/Cas9 generated Ago2 KO cell line, AF525, was derived from exo-siRNA competent embryonic *Ae. aegypti* AF5 cells. Indeed, AF525 cells have been shown to be RNAi deficient with their silencing ability reduced by approximately 50% and have impaired antiviral capabilities against alphaviruses and bunyaviruses which are sensitive to Ago2 action but not ZIKV which is not affected by Ago2 silencing. Thus, these cells can be used to generate cell lines expressing a protein of interest (26, 41). After identification of the putative Ago2 DEDH tetrad we generated a catalytic mutant, where all four of the DEDH residues were mutated, of *Ae. aegypti* Ago2 in an expression construct pPUb-Zeo-2A2A-V5Ago2 we had previously generated (16). For this, AF525 cells were transfected with constructs expressing either the Ago2, a catalytic tetrad negative mutant Ago2 (subsequently called Ago2mut) or eGFP as an experimental control. Transfected cell cultures were grown and passaged in the presence of a selection agent (Zeocin) for approximately 4 weeks to obtain AF525 cells stably expressing V5-tagged Ago2, Ago2mut, or eGFP. The cell lines (AF525-V5-eGFP, AF525-V5-Ago2 and AF525-V5-Ago2mut) generated were screened for the expression of the overexpressed proteins of interest by Western blotting (Fig. 2*A*). Two replicates of each cell line were generated in parallel to ensure the insertion of the genes does not impact the cell growth characteristics. To further characterize the cell lines overexpressing control, Ago2 or Ago2mut the growth of the cultures was monitored by measuring cell density over time. Stable AF525 cells expressing genes of interest were seeded and counted every day for 1 week. No differences in the growth kinetics were observed in the three cell lines at any time point sampled (Fig. 2, *B* and *C*). In addition, there were no discernible growth kinetic differences in between the two batches of stable cell lines, and they were found to stably express similar levels of the proteins of interest throughout multiple passaging (passage 20 shown in Fig. 2*A*). Thus, clone one of each cell line was selected for subsequent analysis.

### The DEDH catalytic tetrad is essential for the antiviral function of Ago2

**Figure 3:**
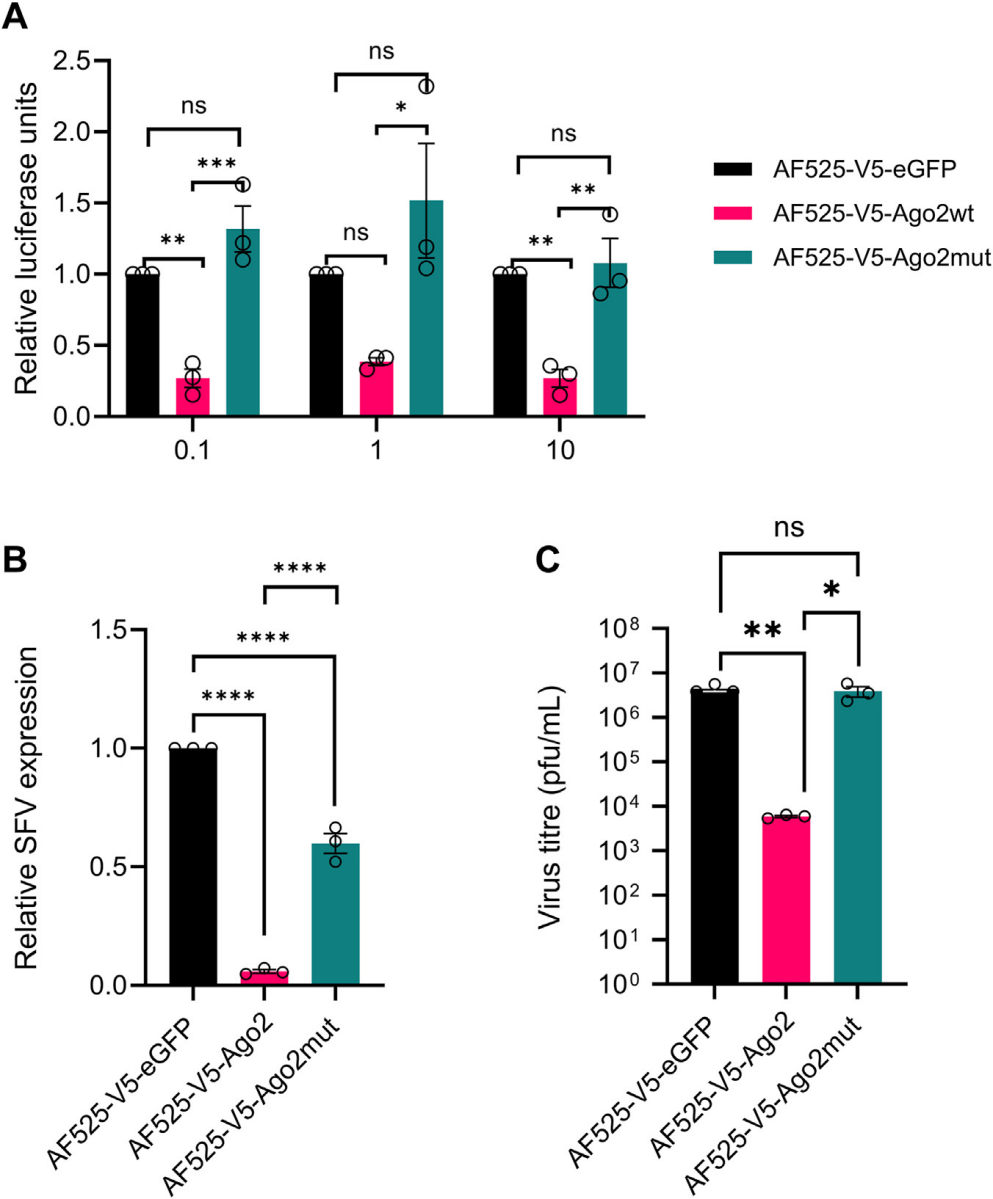
**Effect of catalytic domain mutant on the antiviral activity of Ago2**. AF525 cells stably expressing eGFP (AF525-V5-eGFP; *black*), Ago2 (AF525-V5-Ago2; *pink*) and Ago2mut (AF525-V5-Ago2mut; *teal*) were (*A*) infected with SFV4(3H)-FFLuc at differing MOIs (0.1, 1, and 10 PFU/cell) and virus replication assessed 24 h post-infection (h.p.i.) by measuring FFLuc activity in infected cell monolayers. *B*, infected with wt SFV at a MOI of 1 PFU/cell and relative virus RNA level was assessed at 24 h.p.i. by RT-qPCR (*C*) infected with wt SFV at a MOI of 1 PFU/cell and virus titer was assessed at 24 h.p.i. by plaque assay. Bars show the average ± SEM of three independent experiments performed in triplicate. Significance was determined by one-way ANOVA. Here, ns: non-significant, ∗*p* ≤ 0.05, ∗∗*p* ≤ 0.01, ∗∗∗*p* ≤ 0.001, ∗∗∗∗*p* ≤ 0.0001.

Ago2 is known to be critical for the antiviral activity of the exo-siRNA pathway *in vitro*, moreover mosquitoes without Ago2, or where Ago2 has been experimentally silenced, are more susceptible to arbovirus infection and infection of mosquitoes lacking Ago2 resulting in increased mortality of the arthropod (7, 10, 27, 32, 35). To determine whether the DEDH domain and degradation of the RNA passenger strand are important for the antiviral function of Ago2, AF525 cells stably expressing eGFP, Ago2, or Ago2mut were infected with an alphavirus, Semliki Forest virus (SFV), expressing Firefly luciferase, FFLuc (SFV4(3H)-FFLuc), previously described in (12). FFLuc expression serves as a proxy for the measurement of virus replication. Regardless of the multiplicity of infection (MOI) tested, virus replication was significantly reduced compared to eGFP-expressing control cells in the Ago2-expressing AF525 cells (Fig. 3*A*). In contrast, virus replication in the Ago2mut-expressing cells was not significantly different from the eGFP-expressing control cells. Further, different AF525 cells were infected with SFV at MOI of 1 PFU/cell, and virus replication was measured by RT-qPCR, while infectious virus production was measured by plaque assay. Viral genomic RNA levels were measured and calculated relative to the expression of S7 ribosomal RNA and normalized to the amount of SFV RNA present in the eGFP-expressing cells. Viral genome expression in the SFV-infected Ago2-expressing cells was significantly reduced compared to both the eGFP control and the Ago2mut expressing cells (Fig. 3*B*). A similar result was observed when the virus yield from infected cell cultures was assayed. Virus production was significantly reduced in Ago2-expressing AF525 cells compared to the eGFP control or Ago2mut-expressing cells, with again no significant difference in released virus titer found between the latter two conditions (Fig. 3*C*). We observed a reduction in viral genome copy numbers in Ago2mut cells compared to control cells, although RNA levels are significantly higher than wild-type Ago2 suggesting perhaps some residual activity from another Ago or Ago-related protein such as Ago1. The residual activity did not affect viral gene expression (Fig. 3*A*) or virus titers (Fig. 3*C*) indicating that any residual activity reduction of viral RNA was not strong enough to have an effect.

### The catalytic tetrad is required for exo-siRNA pathway activity

**Figure 4:**
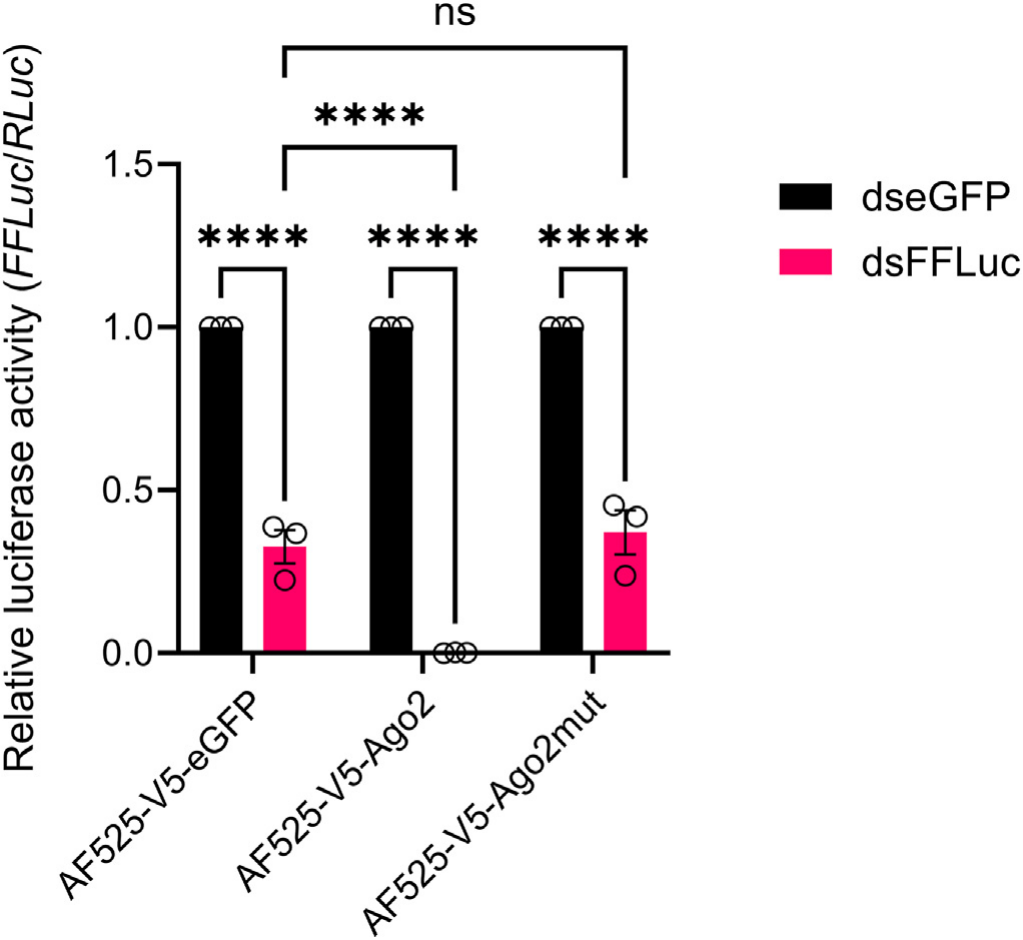
**Effect of the Ago2 catalytic tetrad on exo-siRNA pathway activity**. AF525 cells stably expressing eGFP (AF525-V5-eGFP), Ago2 (AF525-V5-Ago2), and Ago2mut (AF525-V5-Ago2) were transfected with plasmids expressing FFLuc and *Renilla* luciferases and either dsRNA to FFLuc (dsFFLuc; *pink*) or eGFP (dseGFP; *black*). Luciferase activities in each cell line were determined by calculating activity relative to the dseGFP-transfected control. Cells were lysed at 24 h post-transfection (p.t.). Bars show the average of three independent experiments ±SEM performed in triplicate. Significance was assessed by a 2-way ANOVA, ns: non-significant, ∗∗∗∗*p* ≤ 0.0001.

To investigate the role of our Ago2 DEDH mutant in the exo-siRNA pathway, we utilized our previously described exo-siRNA sensor assay (25, 29). The AF525 derived cell lines expressing Ago2, Ago2mut, and eGFP were transfected with plasmids expressing FFLuc and *Renilla* luciferase, as well as dsRNAs targeting FFLuc or eGFP. The silencing abilities of the wild type or mutant Ago2 were determined by measuring the level of FFLuc expression relative to the control *Renilla* luciferase expression. In AF525 cells expressing Ago2 FFLuc expression was reduced by almost 100% when dsFFLuc was transfected compared to the control samples where eGFP dsRNA was used (Fig. 4). In comparison, in the AF525 cells expressing eGFP, FFLuc expression was reduced by around 60% in cells transfected with dsFFLuc compared to dseGFP (Fig. 4). This is in line with previous observations where a small amount of silencing was retained in Ago2 knockout cells (26). Importantly, AF525 cells expressing Ago2mut were comparable to eGFP control where the silencing activity of Ago2 has been lost due to the mutation in the catalytic tetrad (Fig. 4).

### Mutation of the DEDH catalytic tetrad affects the small RNA processing function of Ago2

**Figure 5:**
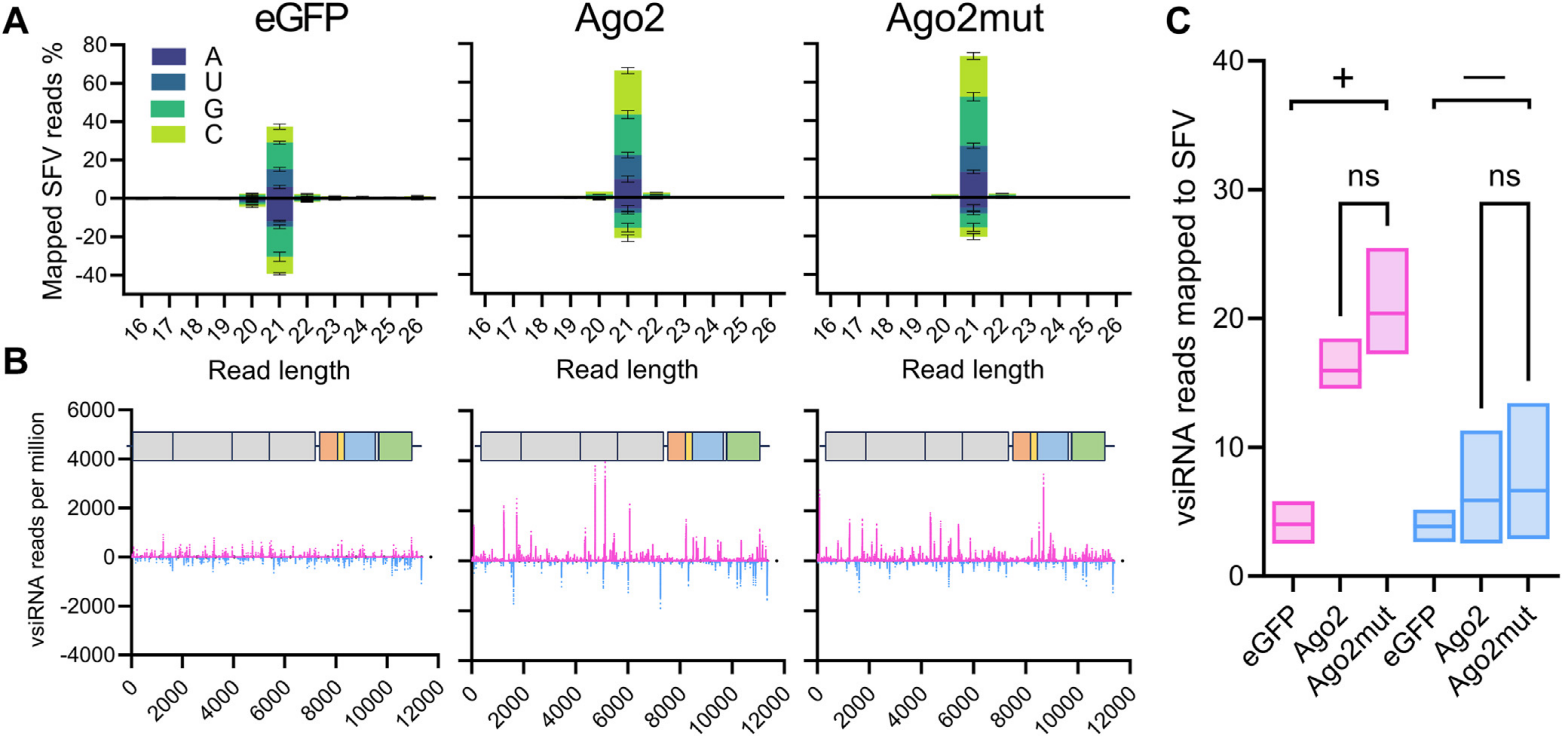
**Mutation in the catalytic tetrad of Ago2 in AF525 cells has a limited effect on the magnitude of 21 nt vsiRNA reads produced in response to SFV infection**. Small RNA analysis of AF525 cells stably expressing eGFP, Ago2, and Ago2mut, and infected with SFV (MOI 5 PFU/cell), analyzed at 24 h.p.i. *A*, histogram of 21 nt vsiRNAs that mapped to the SFV genome (positive) or antigenome (negative) with colors indicating the nt prevalence of the first base for each read length. Shown as mean % mapped reads (Y axis, percentage reads) from three independent experiments. Error bars show the standard error of the mean. *B*, coverage of SFV-derived 21 nt vsiRNAs over the viral genome, genomic (*magenta*) or antigenomic (*cyan*) sense RNAs are shown (Y axis, vsiRNA reads per million) from three independent experiments. Error bars show the standard error of the mean. *C*, mean number of 21 nt viral RNAs per million reads mapping to the SFV genome (*magenta*, +) and antigenome (*cyan*, −) of all treatments. The graph indicates a mean value of three (n = 3) independent repeats with the range of values given. Ordinary one-way ANOVA with Tukey's multiple comparisons test was used to compare groups. Here, ns: insignificant ∗*p* ≤ 0.05, and ∗∗∗*p* ≤ 0.0001. GenBank ID for SFV: KP699763.

To evaluate the influence of the Ago2 catalytic tetrad on small RNA processing, the profiles of vsiRNAs from Ago2, Ago2mut, and eGFP expressing cells were determined. For this, cells were infected with wt SFV at a MOI of 5 PFU/cell for 24 h and immunoprecipitation of V5 tagged Ago2, mutant Ago2, or eGFP was conducted on wt SFV-infected cells. Following immunoprecipitation, RNAs bound to Ago2, Ago2mut, or eGFP were extracted and analyzed by small RNA sequencing. Bioinformatic analysis of small RNA libraries revealed a strong preference for 21 nt small RNA lengths in the Ago2 and Ago2mut libraries (Fig. S2). Examination of vsiRNAs mapping to the SFV genome and antigenome across all treatments (Fig. 5*A*) also indicated a strong bias toward vsiRNA lengths of 21 nt for both the genome and antigenome-derived reads. Coverage mapping of the 21 nt vsiRNAs demonstrated variable distribution patterns of this small RNA across different SFV genomic regions (Fig. 5*B*). Notably, the overall small RNA quantities between the AF525 cell lines expressing Ago2 and Ago2mut cells were comparable, with no statistically significant differences observed either in total or from either sense or antisense genomic strands (Fig. 5, *A* and *C*), which is not unexpected as the cells have functional Dcr2.

**Figure 6:**
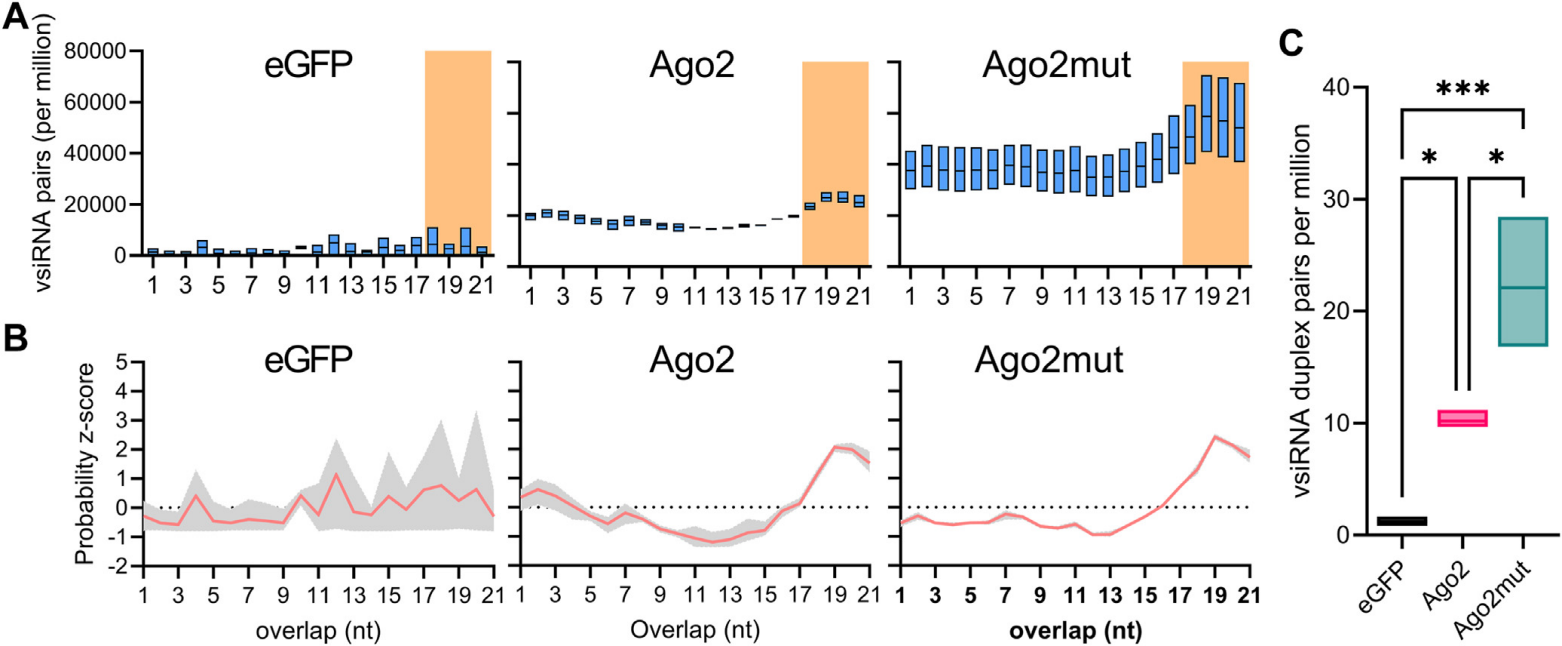
**Mutation of the catalytic tetrad in Ago2 increases the absolute number of SFV-derived vsiRNA duplex pairs**. Samples as described in Figure 5 were reanalysed for bioinformatic analysis. *A*, number of overlapping 21 nt vsiRNA pairs, normalized per million mapped SFV reads, with the most abundant vsiRNA duplex pairs indicated by the orange background as 21 nt reads overlapping by 18-21 nt. *B*, probability z-score of overlapping 21 nt vsiRNA reads across conditions, *e.g.*, eGFP, Ago2 or Ago2mut samples. *C*, number of most abundant 21 nt vsiRNA duplexes overlapping by 18-21 nt across conditions, *e.g.*, eGFP, Ago2, or Ago2mut samples, normalized to number of million mapped reads. All graphs indicate a mean value of three (n = 3) independent repeats with the range of values indicated. Ordinary one-way ANOVA with Tukey's multiple comparisons test was used to compare groups in (*C*) with ∗*p* ≤ 0.05 and ∗∗∗*p* ≤ 0.0001.

The predominant size of siRNAs in mosquito cells is 21 nt in length with a complementary overlap of 19 nt and a 2 nt overhang on each strand. When active, Ago2 will cleave one of the strands termed the “passenger” strand into two molecules of 12 nt and 9 nt in length. While there are limited differences between the absolute numbers of bound small RNAs mapping to the SFV genome or antigenome between Ago2 or Ago2mut proteins (Fig. 5*C*), given the role of the Ago2 catalytic tetrad in the cleavage of the passenger strand of the siRNA duplex into 12 and 9mers (42, 43) we investigated the abundance and composition of vsiRNA duplexes between all treatments. To remove the confounding potential increase in vsiRNAs observed in the AF525 cells expressing Ago2mut we calculated the abundance of 21 nt pairs of reads overlapping between 1 to 21 nt and normalized to the number of million reads in each library. This analysis showed an increased accumulation of virus derived small RNA duplexes across all sizes of overlap for Ago2mut (Fig. 6*A*). Additionally, both the Ago2 and Ago2mut groups demonstrated the highest z-score probabilities for 19 nt overlapping vsiRNA duplexes. The higher z-scores provided increased confidence that the majority of the siRNAs present in the sample are of 19 nt in length with 2 nt overlaps. Notably, the Ago2mut sample displayed a z-score of 2.42, whereas there was a lower z-score of 2.08 for Ago2 sample, indicating a higher probability of 19 nt overlaps with Ago2mut (Fig. 6*B*). Finally, when examining the most abundant 21 nt vsiRNA duplex species, those with overlaps of 18 to 21 nt, we observed a statistically significant increase in the number of vsiRNA duplexes, normalized per million, bound to Ago2mut compared to Ago2 (Fig. 6*C*). The magnitude of this increase is not observed in the SFV specific raw reads (Table S2) indicating this increase is not solely due to increased virus replication in these cells. Collectively the results indicate that the catalytic tetrad mutant Ago2 has a higher proportion of vsiRNA duplexes compared to functional Ago2, and the 21 nt vsiRNA duplexes are more likely to overlap by 19 nt in the Ago2mut expressing cell samples, suggesting that the passenger strands of these vsiRNA duplexes were not being processed into the 9 and 12mer products and duplexes remain intact thus interrupting Ago2 activity.

## Discussion

The exo-siRNA pathway is a critical antiviral control mechanism across the phylum *Arthropoda*. Considering the importance of this pathway, there are still gaps in understanding its regulation, control, and function in mosquitoes. A large proportion of the knowledge has been assumed based on findings observed in the model insect *D. melanogaster* (44). However, there have been important differences observed between studies in model systems such as *D. melanogaster* and vector mosquitoes. For example, the piRNA pathway, an important antiviral response in *Ae*. *aegypti* (9, 16, 22, 23) does not show similar antiviral activity in *D. melanogaster* (45). Had these studies only been performed in model insects, this vital arbovirus control pathway would not be known, limiting our virus control strategies. With technological advances, we are no longer restricted to available tools and reagents, it is now vital that assumptions are assessed in vector species to allow the advancement of arthropod control strategies and improved understanding of arbovirus-vector interactions and transmission.

Our group has identified domains of *Ae. aegypti* Dcr2 that are responsible for siRNA size regulation (46) and vsiRNA production (47), however *Ae. aegypti* Ago2 remains understudied. To overcome this, we identified and investigated the slicing function of Ago2. Recently, Jin *et al.* (2021) reviewed the presence and role of the Argonaute catalytic tetrad, which was shown to be composed of the amino acid motif, DEDX; showing that when the tetrad was absent from Argonaute, the protein lost the reported slicing function (37).

We therefore first sought to determine if this tetrad was found in Ago2 derived from *Ae. aegypti.* We confirmed the presence of the tetrad in three vector species by multiple sequence alignment. Alignment of the Ago2 sequences to *D. melanogaster* Ago2 identified conserved amino acids D1002, E1042, D1074 and H1212 that are in comparable positions within the PIWI domain.

After confirmation of the presence of the tetrad, we next wanted to identify a role for the DEDH tetrad in the function of *Ae. aegypti* Ago2. The DEDH domain was mutated to alanine and cell lines stably expressing the mutant or wild-type Ago2 were generated. Using these cell lines, we showed that by mutating the catalytic tetrad exo-siRNA pathway activity is lost, thus confirming the essential nature of Ago2 and slicing activity in this pathway. Moreover, mutation of the catalytic tetrad resulted in an increase in SFV replication and production of infectious virions. Interestingly, in accordance with the initial observations by Scherer *et al.* (2021), a small level of silencing activity is retained in cells in the absence of Ago2 as determined by silencing assays. The reason for this is still unknown however, the presence of Ago1 or other Ago-related proteins in the cells may account for this observation. However, reintroduction of wild-type Ago2 resolves the silencing impairment and the introduction of the DEDH mutant Ago2 does not have any effect. The residual activity of the siRNA pathway is also observed when we measured virus RNA levels but not when comparing luciferase protein levels or titres. We hypothesize that this may be due to the inherently unstable nature of the RNA, in which small levels of activity are more obvious in contrast to when we measure more stable protein levels.

While we have shown that by mutating the DEDH catalytic tetrad of Ago2 the antiviral and siRNA pathway functions of Ago2 are compromised the effect of catalytic tetrad loss was still to be determined. To this end we performed small RNA sequencing of small RNAs bound to Ago2. The size and distribution of the siRNAs mapping to SFV were not significantly different between the Ago2 wild type and the Ago2 mutant indicating that Dcr2 processing is not affected by the mutation in Ago2, and the functions of both proteins are separate. Interestingly, we observed some siRNAs in the eGFP pulldown fractions which is unexpected. We hypothesize that in the absence of Ago2 SFV replication is high resulting in increased Dcr2 processing of siRNAs. Due to eGFP being a notoriously “sticky” protein it is perhaps unsurprising that some siRNAs either “stick” to eGFP or are carried through the pulldown process due to the increased numbers. Importantly there are at least 3 times more reads associated with Ago2 compared to eGFP. The presence of siRNAs after eGFP pulldown has also been observed previously (16). Importantly, we observed a significant increase in the number of 19 nt vsiRNA duplexes present in the Ago2 DEDH mutant samples in comparison with the functional Ago2. This suggests that even though Dcr2 is producing the small RNAs and Ago2 can bind these, mutant Ago2 is unable to unwind and degrade the passenger strand of the siRNA meaning there is, an accumulation of vsiRNA duplexes. These data confirm the slicing role of the DEDH mutant in *Ae. aegypti* and its critical evolutionary conserved importance in antiviral responses.

## Experimental procedures

### Cell culture

AF525 cells, an Ago2 knockout cell line derived from Aag2-AF5 cells by CRISPR/Cas9 (26, 41) were maintained in Leibovitz's L-15 medium supplemented with 10% fetal bovine serum (Gibco), 10% tryptose phosphate broth (Gibco), and penicillin-streptomycin (final concentration 100 units/ml, 100 μg/ml; Gibco) and maintained at 28 °C. Baby hamster kidney cells-21 (BHK-21 (PHE 85011433)) were cultured in Glasgow modified Eagle's medium (Gibco) supplemented with 10% FBS, 10% tryptose phosphate broth (TPB), and penicillin-streptomycin (final concentration 100 units/ml, 100 μg/ml; Gibco) and maintained at 37 °C with 5% CO_2_.

### Plasmids

pCMV-SFV4(3H)-FFLuc (12, 48), pCMV-SFV4 (49), pPUb-Zeo-2A2A-V5-eGFP (16), pPUb-Zeo-2A2A-V5-Ago2 (16), pGL3-PUb (50), and pPUb-RLuc (29) were used for experiments performed in this study.

### Ago2 mutant generation

A DNA fragment of *Ae. aegypti* Ago2 nucleotides 2089 to 2919 containing D740A, E780A, D812A and H950A mutations was designed and synthesized by Thermo Fisher Scientific. The DEDH mutant fragment was amplified using gene specific primers containing overhangs homologous to the pPUb-Zeo-2A2A-V5-Ago2 vector. In addition, pPUb-Zeo-2A2A-V5-Ago2 was linearized by PCR. Primer sequences can be found in Table S1. All PCRs were performed using Phusion High-Fidelity DNA polymerase (Thermo Fisher Scientific). The PCR products were column purified using PCR purification kit (Qiagen) and DNA concentration was measured using a nanodrop spectrophotometer. The PCR products were digested with DpnI (New England Biolabs) to remove residual plasmid DNA. The digested DNA was column purified using PCR purification kit (Qiagen). The DEDH fragment was joined into the linearized pPUb-Zeo-2A2A-V5Ago2 vector by homologous recombination using In-Fusion Snap Assembly Mastermix (Takara). DH5α bacteria (Thermo Fisher Scientific) were used for transformations following the manufacturer’s instructions. pPUb-Zeo-2A2A-V5-Ago2mut generation was confirmed by sequencing.

### Production of stable cell lines

To produce stable cell lines, one confluent T25 cm^2^ flask of AF525 cells was transfected with 5 μg of the appropriate plasmid linearized with NotI (New England Biolabs) using Dharmafect2 (Horizon Discovery), following the manufacturer's protocol. Duplicate transfections were carried out for each plasmid. At 24 h.p.t., the growth medium was replaced and zeocin was added as a selection marker to a final concentration of 100 μg/ml (InvivoGen). Dead cells were removed, and fresh medium was replaced every 3 or 4 days for approximately 1 month until the cells became confluent. Once confluency was reached cells were passaged in the presence of zeocin at a concentration of 200 μg/ml (InvivoGen).

### dsRNA production

To synthesize dsRNA targeting eGFP and FFLuc, DNA was amplified from pPUb-Zeo-2A2A-V5eGFP and pGL3-PUb using gene-specific primers incorporating the T7 RNA polymerase promoter sequences at the 5′ end. PCR was carried out using KOD Hot Start DNA Polymerase (Novagen). dsRNA was produced using the MEGAscript RNAi kit (Thermo Fisher Scientific) following the manufacturer’s protocol. The resulting purified DNA products were *in vitro* transcribed using T7 RNA polymerase followed by RNAse A and DNase I treatment. The dsRNA was then purified by column purification and concentration was measured using a nanodrop spectrophotometer.

### Virus production and titration

SFV4 and SFV(3H)-FFLuc virus stocks were produced by transfecting pCMV-SFV4 or pCMV-SFV(3H)-FFLuc (12, 48) into BHK-21 cells (PHE 85011433) using DharmaFECT2 reagent (Horizon Discovery). Briefly, approximately 3 × 10^5^ BHK-21 cells per well were plated in 6-well plates and 24 h later transfected with 1 μg of plasmid per well. Three days later once extensive CPE was observed, the supernatant was collected and clarified centrifugation at 2000 rpm for 5 min at 4 °C. The clarified supernatant was then added to a T175 cm^2^ flask containing 80% confluent BHK-21 cells in GMEM containing 2% FBS and 10% TPB and penicillin-streptomycin (final concentration 100 units/ml, 100 μg/ml; Gibco). The cells were incubated at 37 °C for two-three days until extensive CPE was observed, thereafter the supernatant was collected and clarified by centrifugated at 2000 rpm for 20 min at 4 °C. The clarified supernatant was aliquoted and stored at −70 °C. For stock titration *via* plaque assay, BHK-21 cells were plated in 12-well plates at a density of 1.8 × 10^5^ cells/well. After removing medium, 200 μl of 10-fold serial dilutions of the virus stocks were added to the cells. The plates were incubated at 37 °C for 1 h before overlaying with 2 ml of a mixture composed of a 1:1 ratio of 2XMEM containing 4% FBS and 1.2% Avicel. After 48 h incubation 1 ml of 10% formalin (Merck) was added and incubated for 1 h. Cells were stained with 0.1% toluidine blue (Merck) for 20 min.

### Western blotting

Cells were lysed in 1× LDS loading dye (Thermo Fisher Scientific) and 1× reducing agent (Thermo Fisher Scientific) and lysates were stored at −20 °C. Nucleic acid was digested by the addition of benzonase (Merck). Samples were denatured by boiling at 95 °C for 5 min and then run on a 4 to 12% bis-tris gel (Thermo Fisher Scientific). LiCor Chameleon ladder (LiCor) was used to determine molecular weight. Gels were run at 110V for 1.5 h. Protein was transferred onto a nitrocellulose membrane by semi-dry transfer at 15 V for 30 min. The membrane was then blocked in blocking buffer (5% milk-PBS-T) for 1 h and washed in 1× PBS-T 3 times before incubation with the primary antibody [anti-V5 (1:2000) (Abcam ab27671) + and anti-tubulin (1:2000) (Merck T5168)] overnight at 4 °C. The membrane was then washed in 1× PBS-T 3 times before being incubated with LI-COR fluorescent secondary antibody (goat anti-mouse IgG (1:5000) (LI-COR)) for 1 h at RT. Finally, the membrane was washed in 1× PBS-T twice followed by a final wash with water. Proteins were visualized on a LI-COR Odyssey DLx Imaging system.

### RNAi reporter assays

AF525-V5-eGFP, AF525-V5-Ago2 and AF525-V5-Ago2mut cells were seeded at a density of 2.3 × 10^5^ cells per well in a 24-well plate. After 24 h cells were co-transfected with 50 ng of pPUb-FFLuc and 20 ng pPUb-RLuc and 20 ng of dsRNA targeting either FFLuc or eGFP as control. Transfection was carried out with Dharmafect2 (Horizon Discovery) following the manufacturer’s instructions. At 24 h.p.t. cells were lysed in 1× Passive Lysis Buffer (Promega) and luciferase activities were measured using a Dual-Luciferase Reporter Assay System (Promega).

### Small RNA immunoprecipitation assay (IP) and RNA extraction

Cells expressing eGFP, Ago2 or Ago2mut (AF525-V5-eGFP, AF525-V5-Ago2 and AF525-V5-Ago2mut cells described earlier) were seeded at a density of 7.6 × 10^6^ cells per T25 cm^2^ flask. 24 h after plating the cells were infected with SFV4 at a MOI of 5. At 24 h.p.i., the cells were pelleted and lysed in 700 μl of IP lysis buffer (20 mM Tris pH 7.5, 150 mM NaCl, 5 mM MgCl_2_, 0.5% NP-40 with protease inhibitors [*cOmplete EDTA*-*free tablets*, *Roche*] and phosphatase inhibitors [*PhosSTOP*, *Roche*]). Lysates were centrifuged at 16000g for 20 min. 50 μl supernatant was removed and stored at −20 °C for confirming protein expression (before immunoprecipitation) by immunoblotting. The remaining supernatant was transferred to tubes containing 20 μl of protein G beads coupled to V5 antibody resuspended in IP lysis buffer and rotated at 4 °C overnight. The following day, the proteins were washed three times with wash buffer (50 mM Tris pH 7.5, 200 mM NaCl, 1 mM EDTA, 1% NP-40 with protease inhibitors [*cOmplete EDTA*-*free tablets*, *Roche*]) and eluted with 60 μl of 100 mM TEAB + 5% SDS. 10 μl of the eluted sample was stored for western blotting. Following immunoprecipitation, the eluate was treated with 5 μl of proteinase K (Merck) (20 mg/ml) and incubated for 1 h at 50 °C to remove all protein. 1 ml of TRIzol reagent (Thermo Fisher Scientific) was added to the eluted sample and processed according to the manufacturer’s instructions and then precipitated using glycogen as a carrier.

### RNA extraction from cells

Cells were lysed in 1 ml TRIzol (Thermo Fisher Scientific) and total RNA extraction was performed as described in the manufacturer’s protocol. RNA concentrations were measured by nanodrop spectrophotometer.

### cDNA synthesis

cDNA synthesis from extracted RNA was performed using SuperScript III (Thermo Fisher Scientific) and oligo(dT)_15_ primer (Promega) as described in the manufacturer's instructions.

### qPCR analysis

qPCR was performed on transcribed cDNA using SYBR green mastermix (Thermo Fisher Scientific) on an Applied Biosystems Quant Studio three machine. MIQE guidelines for qPCR can be found in https://doi.org/10.5525/gla.researchdata.1795.

### Small RNA library construction and sequencing

Small RNA library construction and sequencing were undertaken at BGI (Beijing Genomics Institute) using their Small RNA Library Construction Protocol (DNBSEQ). Briefly, around 1 μg isolated total RNA from each sample was size selected for 15 to 40 nt *via* PAGE gel. The size-selected sRNA then underwent 3′ adaptor ligation (70 °C, 2 min; ice for 1 min; 25 °C, 2 h; held at 4 °C), followed by 5′ adaptor ligation (70 °C, 2 min; ice for 1 min; 25 °C, 1 h; held at 4 °C). Reverse transcription was then conducted by adding RT-Primer to the reaction solution (65 °C, 3 min; ice for 1 min). A Reverse Transcription Reaction Mixture, consisting of FS Reaction Buffer Mix, RNase Inhibitor, and RT Enzyme, was prepared on ice and added to the tube (42 °C, 60 min; 70 °C, 15 min). The reaction was then held at 4 °C. cDNA amplification utilized a PCR Primer Mix, with an initial denaturation at 95 °C for 3 min (1 cycle), followed by 16–17 cycles of amplification (98 °C for 20 s, 56 °C for 15 s, 72 °C for 15 s), and a final extension at 72 °C for 10 min. The reaction was then held at 4 °C. The PCR products were purified using PAGE gel, dissolved in EB solution, and circularized to form ssCir DNA libraries. The final prepped libraries were assessed with an Agilent Technologies Fragment bioanalyzer and sequenced on a DNBSEQ-G400 instrument, configured for 50 bases single-end read.

### Small RNA sequencing and analysis

Basecalled fastq files were quality and adapter trimmed using the fastp tool (v0.23.2) (51) retaining 16 to 30 nt reads under default conditions. The clean, trimmed reads were then mapped to the SFV genome (GenBank ID: KP699763) using Bowtie2 (v2.4.5) (52), with the sensitive mapping flag (—sensitive). Trimming and mapping statistics summary is available in Table S2. The histogram of mapped read lengths and first base pair bias was generated using viral_sRNA_tools/3_bam_sRNA_histogram.sh script, which utilizes samtools (v1.16.1).

Output BAM files from Bowtie2 were filtered to include only the 21 nt reads. Coverage statistics for each position of the SFV genome were extracted using the bedtools genome coverage tool (v.2.27.1) (53) and visualized using GraphPad Prism (v10.0.2). To determine overlapping vsiRNA pairs, overlapping pairs of 21 nt reads and their overlap probabilities (z-score) were calculated using the small RNA signatures Python script signature.py (54), with the following conditions (--minquery 21 --maxquery 21 --mintarget 21 --maxtarget 21 --minscope 1 --maxscope 21), read pairs were then normalized to the number of million reads per library (54).

### Data analyses

Viral small RNA metrics, coverage, and overlapping pairs analysis were visualized using GraphPad Prism (v10.0.2). All graphs and statistical analyses were prepared using GraphPad Prism (v.10.0.2).

## Data availability

Data underlying figures are available under https://doi.org/10.5525/gla.researchdata.1795. Small RNA sequencing generated for this study have been deposited in the NCBI Sequence Read Archive (SRA), available under accession number PRJNA1057508. Scripts utilized for the small RNA analysis are available from in the viral_sRNA_tools GitHub repository at https://github.com/rhparry/viral_sRNA_tools.

## Supporting information

This article contains supporting information. References for primer sequences contained in Table S1 are (10, 55, 56).

## Conflict of interest

The authors declare that they have no conflicts of interest with the contents of this article.

## References

[1] Tikhe C.V., Dimopoulos G. (2021). Mosquito antiviral immune pathways. Dev. Comp. Immunol..

[2] Blair C.D., Olson K.E. (2015). The role of RNA interference (RNAi) in arbovirus-vector interactions. Viruses.

[3] Olson K.E., Blair C.D. (2015). Arbovirus-mosquito interactions: RNAi pathway. Curr. Opin. Virol..

[4] Donald C.L., Kohl A., Schnettler E. (2012). New insights into control of arbovirus replication and spread by insect RNA interference pathways. Insects.

[5] Bronkhorst A.W., van Rij R.P. (2014). The long and short of antiviral defense: small RNA-based immunity in insects. Curr. Opin. Virol..

[6] Samuel G.H., Adelman Z.N., Myles K.M. (2018). Antiviral immunity and virus-mediated antagonism in disease vector mosquitoes. Trends Microbiol..

[7] Keene K.M., Foy B.D., Sanchez-Vargas I., Beaty B.J., Blair C.D., Olson K.E. (2004). RNA interference acts as a natural antiviral response to O'nyong-nyong virus (Alphavirus; Togaviridae) infection of Anopheles gambiae. Proc. Natl. Acad. Sci. U. S. A..

[8] Myles K.M., Wiley M.R., Morazzani E.M., Adelman Z.N. (2008). Alphavirus-derived small RNAs modulate pathogenesis in disease vector mosquitoes. Proc. Natl. Acad. Sci. U. S. A..

[9] Schnettler E., Donald C.L., Human S., Watson M., Siu R.W.C., McFarlane M. (2013). Knockdown of piRNA pathway proteins results in enhanced Semliki Forest virus production in mosquito cells. J. Gen. Virol..

[10] McFarlane M., Arias-Goeta C., Martin E., O'Hara Z., Lulla A., Mousson L. (2014). Characterization of Aedes aegypti innate-immune pathways that limit Chikungunya virus replication. PLoS Negl. Trop. Dis..

[11] Campbell C.L., Keene K.M., Brackney D.E., Olson K.E., Blair C.D., Wilusz J. (2008). Aedes aegypti uses RNA interference in defense against Sindbis virus infection. BMC Microbiol..

[12] Varjak M., Dietrich I., Sreenu V.B., Till B.E., Merits A., Kohl A. (2018). Spindle-E acts antivirally against alphaviruses in mosquito cells. Viruses.

[13] Morazzani E.M., Wiley M.R., Murreddu M.G., Adelman Z.N., Myles K.M. (2012). Production of virus-derived ping-pong-dependent piRNA-like small RNAs in the mosquito soma. PLoS Pathog..

[14] Carissimo G., Pondeville E., McFarlane M., Dietrich I., Mitri C., Bischoff E. (2015). Antiviral immunity of Anopheles gambiae is highly compartmentalized, with distinct roles for RNA interference and gut microbiota. Proc. Natl. Acad. Sci. U. S. A..

[15] Waldock J., Olson K.E., Christophides G.K. (2012). Anopheles gambiae antiviral immune response to systemic O'nyong-nyong infection. PLoS Negl. Trop. Dis..

[16] Varjak M., Maringer K., Watson M., Sreenu V.B., Fredericks A.C., Pondeville E. (2017). Aedes aegypti Piwi4 is a noncanonical PIWI protein involved in antiviral responses. mSphere.

[17] Sanchez-Vargas I., Scott J.C., Poole-Smith B.K., Franz A.W.E., Barbosa-Solomieu V., Wilusz J. (2009). Dengue virus type 2 infections of Aedes aegypti are modulated by the mosquito's RNA interference pathway. PLos Pathog..

[18] Samuel G.H., Wiley M.R., Badawi A., Adelman Z.N., Myles K.M. (2016). Yellow fever virus capsid protein is a potent suppressor of RNA silencing that binds double-stranded RNA. Proc. Natl. Acad. Sci. U. S. A..

[19] Scott J.C., Brackney D.E., Campbell C.L., Bondu-Hawkins V., Hjelle B., Ebel G.D. (2010). Comparison of dengue virus type 2-specific small RNAs from RNA interference-competent and -incompetent mosquito cells. PLoS Negl. Trop. Dis..

[20] Franz A.W., Sanchez-Vargas I., Adelman Z.N., Blair C.D., Beaty B.J., James A.A. (2006). Engineering RNA interference-based resistance to dengue virus type 2 in genetically modified Aedes aegypti. Proc. Natl. Acad. Sci. U. S. A..

[21] Varjak M., Donald C.L., Mottram T.J., Sreenu V.B., Merits A., Maringer K. (2017). Characterization of the Zika virus induced small RNA response in Aedes aegypti cells. PLoS Negl. Trop. Dis..

[22] Dietrich I., Jansen S., Fall G., Lorenzen S., Rudolf M., Huber K. (2017). RNA interference restricts Rift Valley fever virus in multiple insect systems. mSphere.

[23] Dietrich I., Shi X., McFarlane M., Watson M., Blomström A.L., Skelton J.K. (2017). The antiviral RNAi response in vector and non-vector cells against orthobunyaviruses. PLoS Negl. Trop. Dis..

[24] Leger P., Lara E., Jagla B., Sismeiro O., Mansuroglu Z., Coppée J.Y. (2013). Dicer-2- and Piwi-mediated RNA interference in Rift Valley fever virus-infected mosquito cells. J. Virol..

[25] McFarlane M., Almire F., Kean J., Donald C.L., McDonald A., Wee B. (2020). The Aedes aegypti domino ortholog p400 regulates antiviral exogenous small interfering RNA pathway activity and ago-2 expression. mSphere.

[26] Scherer C., Knowles J., Sreenu V.B., Fredericks A.C., Fuss J., Maringer K. (2021). An Aedes aegypti-derived Ago2 knockout cell line to investigate arbovirus infections. Viruses.

[27] Dong S., Dimopoulos G. (2023). Aedes aegypti Argonaute 2 controls arbovirus infection and host mortality. Nat. Commun..

[28] Leggewie M., Scherer C., Altinli M., Gestuveo R.J., Sreenu V.B., Fuss J. (2023). The Aedes aegypti RNA interference response against Zika virus in the context of co-infection with dengue and chikungunya viruses. PLoS Negl. Trop. Dis..

[29] Alexander A.J.T., Salvemini M., Sreenu V.B., Hughes J., Telleria E.L., Ratinier M. (2023). Characterisation of the antiviral RNA interference response to Toscana virus in sand fly cells. PLoS Pathog..

[30] Samuel G.H., Pohlenz T., Dong Y., Coskun N., Adelman Z.N., Dimopoulos G. (2023). RNA interference is essential to modulating the pathogenesis of mosquito-borne viruses in the yellow fever mosquito Aedes aegypti. Proc. Natl. Acad. Sci. U. S. A..

[31] Altinli M., Leggewie M., Schulze J., Gyanwali R., Badusche M., Sreenu V.B. (2023). Antiviral RNAi response in Culex quinquefasciatus-derived HSU cells. Viruses.

[32] Besson B., Lezcano O.M., Overheul G.J., Janssen K., Spruijt C.G., Vermeulen M. (2022). Arbovirus-vector protein interactomics identifies Loquacious as a co-factor for dengue virus replication in Aedes mosquitoes. PLoS Pathog..

[33] Williams A.E., Sanchez-Vargas I., Reid W.R., Lin J., Franz A.W.E., Olson K.E. (2020). The antiviral small-interfering RNA pathway induces Zika virus resistance in transgenic Aedes aegypti. Viruses.

[34] Sucupira P.H.F., Ferreira Á.G.A., Leite T.H.J.F., de Mendonça S.F., Ferreira F.V., Rezende F.O. (2020). The RNAi pathway is important to control mayaro virus infection in Aedes aegypti but not for wolbachia-mediated protection. Viruses.

[35] Sasaki T., Kuwata R., Hoshino K., Isawa H., Sawabe K., Kobayashi M. (2017). Argonaute 2 suppresses Japanese encephalitis virus infection in Aedes aegypti. Jpn. J. Infect. Dis..

[36] Merkling S.H., Crist A.B., Henrion-Lacritick A., Frangeul L., Couderc E., Gausson V. (2023). Multifaceted contributions of Dicer2 to arbovirus transmission by Aedes aegypti. Cell Rep..

[37] Jin S., Zhan J., Zhou Y. (2021). Argonaute proteins: structures and their endonuclease activity. Mol. Biol. Rep..

[38] Joshua-Tor L. (2006). The argonautes. Cold Spring Harb. Symp. Quant Biol..

[39] Pak M.A., Markhieva K.A., Novikova M.S., Petrov D.S., Vorobyev I.S., Maksimova E.S. (2023). Using AlphaFold to predict the impact of single mutations on protein stability and function. PLoS One.

[40] Abramson J., Adler J., Dunger J., Evans R., Green T., Pritzel A. (2024). Accurate structure prediction of biomolecular interactions with AlphaFold 3. Nature.

[41] Fredericks A.C., Russell T.A., Wallace L.E., Davidson A.D., Fernandez-Sesma A., Maringer K. (2019). Aedes aegypti (Aag2)-derived clonal mosquito cell lines reveal the effects of pre-existing persistent infection with the insect-specific bunyavirus Phasi Charoen-like virus on arbovirus replication. PLoS Negl. Trop. Dis..

[42] Rand T.A., Petersen S., Du F., Wang X. (2005). Argonaute2 cleaves the anti-guide strand of siRNA during RISC activation. Cell.

[43] Leuschner P.J., Ameres S.L., Kueng S., Martinez J. (2006). Cleavage of the siRNA passenger strand during RISC assembly in human cells. EMBO Rep..

[44] Blair C.D. (2011). Mosquito RNAi is the major innate immune pathway controlling arbovirus infection and transmission. Future Microbiol..

[45] Petit M., Mongelli V., Frangeul L., Blanc H., Jiggins F., Saleh M.C. (2016). piRNA pathway is not required for antiviral defense in Drosophila melanogaster. Proc. Natl. Acad. Sci. U. S. A..

[46] Reuter M., Parry R.H., McDonald M., Gestuveo R.J., Arif R., Khromykh A.A. (2024). The PAZ domain of Aedes aegypti Dicer 2 is critical for accurate and high-fidelity size determination of virus-derived small interfering RNAs. RNA.

[47] Gestuveo R.J., Parry R., Dickson L.B., Lequime S., Sreenu V.B., Arnold M.J. (2022). Mutational analysis of Aedes aegypti Dicer 2 provides insights into the biogenesis of antiviral exogenous small interfering RNAs. PLoS Pathog..

[48] Rodriguez-Andres J., Rani S., Varjak M., Chase-Topping M.E., Beck M.H., Ferguson M.C. (2012). Phenoloxidase activity acts as a mosquito innate immune response against infection with Semliki Forest virus. PLoS Pathog..

[49] Ulper L., Sarand I., Rausalu K., Merits A. (2008). Construction, properties, and potential application of infectious plasmids containing Semliki Forest virus full-length cDNA with an inserted intron. J. Virol. Methods.

[50] Anderson M.A., Gross T.L., Myles K.M., Adelman Z.N. (2010). Validation of novel promoter sequences derived from two endogenous ubiquitin genes in transgenic Aedes aegypti. Insect Mol. Biol..

[51] Chen S., Zhou Y., Chen Y., Gu J. (2018). fastp: an ultra-fast all-in-one FASTQ preprocessor. Bioinformatics.

[52] Langmead B., Salzberg S.L. (2012). Fast gapped-read alignment with Bowtie 2. Nat. Methods.

[53] Quinlan A.R., Hall I.M. (2010). BEDTools: a flexible suite of utilities for comparing genomic features. Bioinformatics.

[54] Antoniewski C. (2014). Computing siRNA and piRNA overlap signatures. Methods Mol. Biol..

[55] Breakwell L., Dosenovic P., Karlsson Hedestam G.B., D'Amato M., Liljeström P., Fazakerley J. (2007). Semliki Forest virus nonstructural protein 2 is involved in suppression of the type I interferon response. J. Virol..

[56] Donald C.L., Varjak M., Aguiar E.R.G.R., Marques J.T., Sreenu V.B., Schnettler E. (2018). Antiviral RNA interference activity in cells of the predatory mosquito, Toxorhynchites amboinensis. Viruses.

