# Supplementary material for: The catalytic tetrad of Aedes aegypti argonaute 2 is critical for the antiviral activity of the exogenous siRNA pathway
Source: J Biol Chem. 2025 Feb 19;301(4):108332. doi: 10.1016/j.jbc.2025.108332 (PMC11968273; doi:10.1016/j.jbc.2025.108332)
Supplement: Supplementary Figure [file mmc1.docx]

**The catalytic tetrad of *Aedes aegypti* Argonaute 2 is critical for the antiviral activity of the exogenous siRNA pathway.**

Krittika Dummunee^1^, Rhys H. Parry^2^, Lars Redecke^3,4^, Margus Varjak^1,5^, Benjamin Brennan^1*^, Alain Kohl^1,6*^**,** Melanie McFarlane^1*^

**
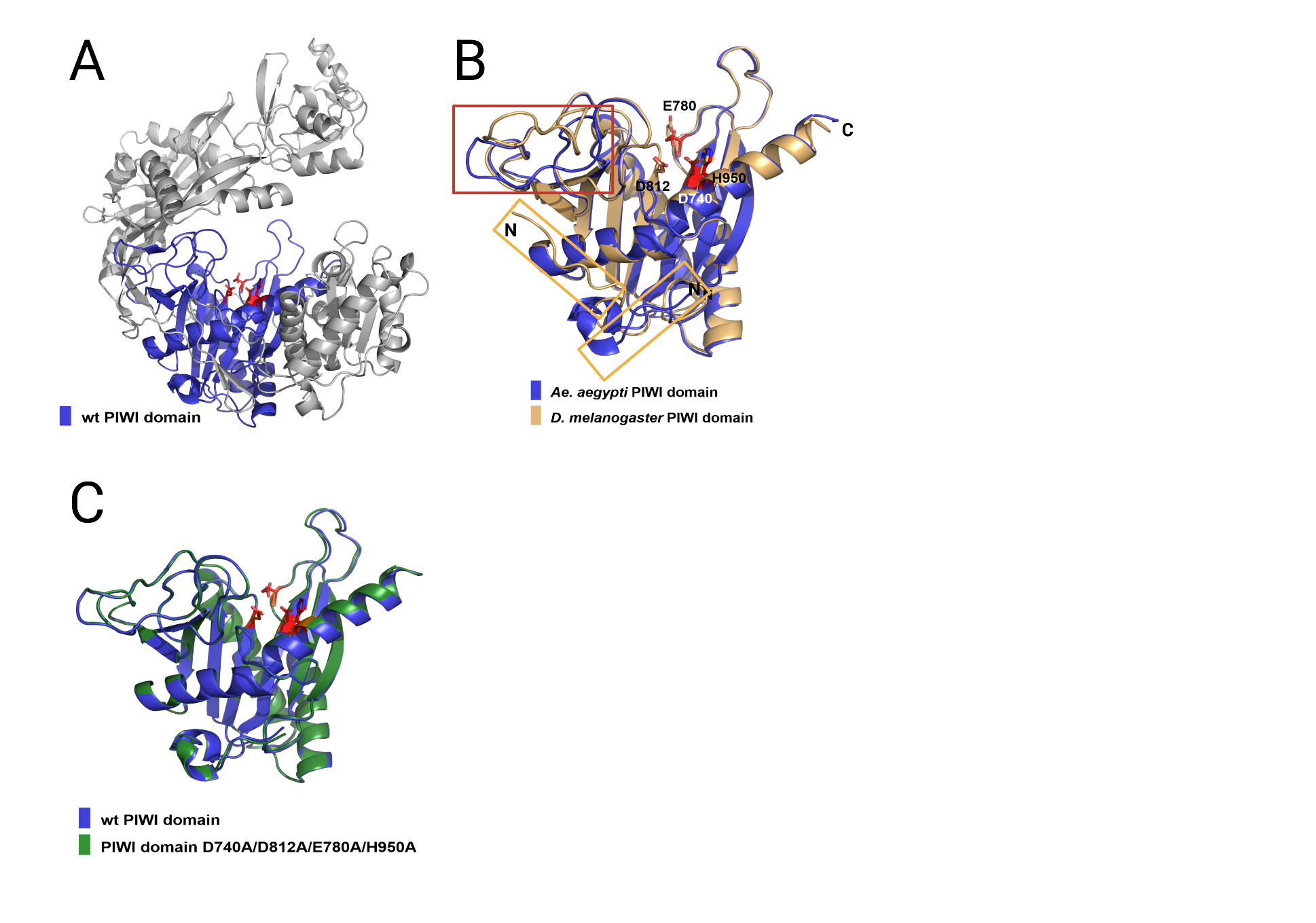
**

**Supplementary Fig. 1. Structure prediction of Ago2 from *Ae. aegypti* and superposition of the wt Ago2 PIWI domain from *Ae. aegypti* and *D. melanogaster*.** **A)** Cartoon representation of the AlphaFold3 calculated three-dimensional structure of wt Ago2 from *Ae. aegypti* (UniProt ID: C5J0H4). Since the prediction of the N terminal residues 1 – 146 is of low confidence (plDDT < 50), indicating high flexibility of the N terminus of Ago2, the presented model only comprises residues 147 – 992 that are suggested to be well-folded as highlighted by a predicted template modelling (pTM) score of 0.93. The PIWI domain is coloured in blue with the residues composing the catalytic tetrad (residues D740, E780, D812 and H950) shown in stick representation (red). **B)** Cartoon representation of the AlphaFold3 predicted three-dimensional structures of the wt Ago2 PIWI domain from *Ae. aegypti* (residues 719-963, UniProt ID: C5J0H4) and *D. melanogaster* (residues 947-1189, UniProt ID: Q9VUQ5). The predicted template modelling (pTM) score of 0.94 obtained for both structural models indicates high confidence. The structures were superposed and visualized using PyMOL Molecular Graphics System. An RMSD of 0.527 Å was calculated for 216 homologous Cα atoms, revealing structural conservation. Larger deviations are limited to a flexible loop area (red box) and the flexible N termini (yellow boxes). The positions of the four residues in the RNAse H fold composing the catalytic DEDH tetrad (stick representation, labelled following *Ae. aegypti* numbering) remain highly conserved in the Ago2 proteins of the two species. **C)** Superposition of the predicted three-dimensional structures of the wt Ago2 PIWI domain (blue, residues 719-963) and the corresponding D740A/E780A/D812A/H950A mutant (green). The pTM scores of 0.94 obtained for both predictions indicate high confidence. The structures were superposed and visualized using PyMOL Molecular Graphics System. An RMSD of 0.162 Å calculated for 214 homologous Cα atoms reveals almost identical structures. The four residues in the RNAse H fold composing the catalytic DEDH tetrad are labelled and shown in stick representation.

**Supplementary Fig. 2.** **Size distribution of the raw small RNA sequencing data and first position base bias represented as total percentage of library.** Each treatment is the average of three samples between the range 16-26 nt and plotted as mean ± SD.
