# Supplementary material for: The catalytic tetrad of Aedes aegypti argonaute 2 is critical for the antiviral activity of the exogenous siRNA pathway
Source: J Biol Chem. 2025 Feb 19;301(4):108332. doi: 10.1016/j.jbc.2025.108332 (PMC11968273; doi:10.1016/j.jbc.2025.108332)
Supplement: Supplementary table [file mmc2.docx]

**Supplementary Table 1. Table of primer sequences.**

| Primer | Sequence 5’-3’ | Use | Ref. |
| --- | --- | --- | --- |
| Ago2 DEDH mutant F | GAGGCGATATGAGCACCATCAGCAATATTTGGCTC | cloning |  |
| Ago2 DEDH mutant R | CTCCTTGGTCAGGTTGTTCATGTTCAGAGGACGATC |  |  |
| Ago2 linearisation F | AACCTGACCAAGGAGTACGAG |  |  |
| Ago2 linearisation R | TGCTCATATCGCCTCGTTTC |  |  |
| T7-eGFP F | **GTAATACGACTCACTATAGGG**GGCGTGCAGTGCTTCAGCCGC | dsRNA | [10] |
| T7-eGFP R | **GTAATACGACTCACTATAGGG**GTGGTTGTCGGGCAGCAGCAC |  |  |
| T7-FFLuc F | **GTAATACGACTCACTATAGGG**ACTTACGCTGAGTACTTC |  | [56] |
| T7-FFLuc R | **GTAATACGACTCACTATAGGG**GAAATCCCTGGTAATCCG |  |  |
| qS7 F | CCAGGCTATCCTGGAGTTG | qRT-PCR | [10] |
| qS7 R | GACGTGCTTGCCGGAGAAC |  |  |
| qSFV F | GCAAGAGGCAAACGAACAGA |  | [55] |
| qSFV R | GGGAAAAGATGAGCAAACCA |  |  |

All primer sequences are shown reading from 5’ to 3’. Bases in bold indicate the T7 promoter sequence.

**Supplementary Table 2.** Small RNA library metadata and mapping analysis.

| **SRA accession** | **Sample** | **Reads in library** | **After trimming** | **Mapping to SFV** | **All %** | **21nt Mapping** | **21nt %** |
| --- | --- | --- | --- | --- | --- | --- | --- |
| SRR27355011 | eGFP1 | 12682605 | 11425388 | 545352 | 4.77 | 364996 | 3.19 |
| SRR27355010 | eGFP2 | 12621900 | 11739371 | 749741 | 6.39 | 618588 | 5.27 |
| SRR27355009 | eGFP3 | 12758264 | 12102302 | 1279654 | 10.57 | 1029924 | 8.51 |
| SRR27355014 | Ago2wt1 | 12728091 | 12667379 | 3700056 | 29.21 | 3179842 | 25.10 |
| SRR27355013 | Ago2wt2 | 12622513 | 12514397 | 2173597 | 17.37 | 1914301 | 15.30 |
| SRR27355012 | Ago2wt3 | 12813315 | 12769948 | 2329461 | 18.24 | 2044492 | 16.01 |
| SRR27355017 | Ago2mut1 | 12667799 | 12661054 | 4916373 | 38.83 | 4601643 | 36.34 |
| SRR27355016 | Ago2mut2 | 12769114 | 12750445 | 2722375 | 21.35 | 2566222 | 20.13 |
| SRR27355015 | Ago2mut3 | 12762643 | 12754776 | 2541042 | 19.92 | 2393148 | 18.76 |
